# Protective effects of turmerosaccharides rich extract of *Curcuma longa* L. in osteoarthritic dogs

**DOI:** 10.3389/fvets.2026.1536366

**Published:** 2026-03-18

**Authors:** S. D. Tripathi, S. Suresh, Prakash Khangal, M Sasi Kumar, H. S. Nandini

**Affiliations:** 1 Department of Surgery and Radiology, Mumbai Veterinary College, Animal and Fishery Sciences University, Nagpur, Maharashtra, India; 2 Department of Pharmacology & Toxicology, R&D Centre, Natural Remedies Private Limited, Bengaluru, Karnataka, India

**Keywords:** canine osteoarthritis, chondroitin sulfate, *Curcuma longa*, glucosamine, joint pain, orthoron, stiffness, Turmacin®

## Abstract

**Introduction:**

Osteoarthritis (OA) is a progressive degenerative joint disease characterized by cartilage deterioration, leading to chronic pain and impaired immobility particularly in older dogs.

**Aim:**

This study investigated the protective effects of a turmerosaccharides-rich extract of *Curcuma longa* L. in the management of canine OA.

**Methodology:**

Twenty dogs with OA were randomized into two treatment groups. One group received the *C. longa* extract orally at 300 mg/20 kg body weight once daily for 42 days. The second group received glucosamine hydrochloride with chondroitin sulfate (Glu/CS), administered orally for the same duration, according to the manufacturer’s recommendations. Therapeutic response was evaluated using laboratory parameters, veterinarian, owner reported assessments, and radiological examination of the affected joints. Dogs were re-evaluated on days 49 and 56 following treatment withdrawal.

**Results and discussion:**

Both veterinarian and owner assessments demonstrated that the *C. longa* extract produced a significant reduction in pain and clinical signs associated with OA. The extract was palatable and well tolerated, with no adverse events observed throughout the study. Radiographic evaluation revealed no substantial changes in joint space or structural integrity. Collectively, these findings demonstrate turmerosaccharide-rich *C. longa* extract is a safe, well-accepted and clinically effective therapeutic option for the management of OA in dogs.

## 1 Introduction

Osteoarthritis (OA) is a chronic, degenerative joint disorder characterized by progressive deterioration of cartilage, periarticular soft tissues and subchondral bone (1). This structural degradation results in pain, reduced joint function and immobility. OA is highly prevalent in the canine population, affecting approximately 20% of dogs at one year of age and up to 90% of older dogs (2). Dogs suffering from OA typically exhibit symptoms such as lameness, joint stiffness, functional disability, and a substantial decline in quality of life.

The underlying pathophysiology of OA is driven by a disruption of the balance between degradation and repair (3). Release of proinflammatory cytokines including TNFα, IL-1, and IL-6 at the joint surface, play a central role in this imbalance by promoting cartilage matrix breakdown and sustaining joint inflammation (4).

A common practice for managing OA in dogs involves the use of Non-Steroidal Anti-Inflammatory. Drugs (NSAIDs). However, their long-term use is limited by the adverse effects, particularly gastrointestinal ulceration (5). Glucosamine and chondroitin sulfate have garnered significant, attention as alternative therapies. Glucosamine, an amino-monosaccharide precursor essential for. glycosaminoglycan (GAG), contributes to maintenance of articular cartilage integrity (1). Chondroitin sulfate, a major component of connective tissues including cartilage, tendons, bones, and vertebral discs (6), acts as a shock absorber, facilitates water retention in cartilage, supports healthy synovial fluid homeostasis, stabilizes collagen fibrils and enhances chondrocyte GAG synthesis. Despite their benefits, both glucosamine and chondroitin sulfate have been associated with gastrointestinal adverse effects such as nausea, vomiting and diarrhoea. Furthermore, glucosamine also causes allergic reactions and nephrotoxicity in some dogs (7).

Given the limitations, *C. longa* (a member of the Zingiberaceae family) presents a potential therapeutic alternative for OA therapy due to broad spectrum of reported pharmacological activities (8). *C. longa* has been documented to possess a broad spectrum of bioprotective functions, including antioxidant, antidiabetic, anti-mutagenic, anticoagulant, anticancer, antifertility, antifungal and antibacterial properties of *C. longa* extracts (9), while additional evidence highlights multiple pharmacological actions of its polar extract, including antitumor, antidiabetic, antimicrobial, hepatoprotective, antifertility, antidepressant, antioxidant, anti-inflammatory and immunomodulatory effects (10–17).

The investigational product evaluated in the present study is a standardized aqueous extract of *C. longa* (Turmacin®/Orthoron), enriched with turmerosaccharides (>10% w/w) with a negligible amount of curcuminoids. Prior *in vitro* and *in vivo* safety and efficacy studies demonstrated significant anti-inflammatory activity of this extract in both acute and chronic models (18). Fractionation studies identified a turmerosaccharide-rich fraction as the principal contributor to the anti-inflammatory and anti-arthritic activity, with greater efficacy than turmerosaccharide-poor. fractions (19, 20). In a comparative study with curcuminoids, Turmacin® exhibited superior efficacy with a rapid onset of osteoarthritic pain relief (21). Clinical studies in humans further demonstrated 1,000 mg of Turmacin® for 42 days reduce pain in OA (22, 23). Safety assessments indicate a favorable toxicological profile, the LD50 of Turmacin® was found to be >5,000 mg/kg rat body weight in an acute oral toxicity study (24), and the No-Observed-Adverse-Effect Level (NOAEL) was determined to be 1,000 mg/kg rat body weight in a sub-chronic oral toxicity study (25).

Based on these compelling preclinical and clinical findings, which strongly support the anti-arthritic effect of the *C. longa* extract, the present study was designed to evaluate its protective effects in dogs with naturally occurring OA. The results of this study were compared with a marketed product containing glucosamine and chondroitin sulfate to benchmark its efficacy.

## 2 Materials and methods

### 2.1 Investigational product (IP)

The investigational product (NR-INF-02; Turmacin®) is an aqueous extract derived from the rhizome of *Curcuma longa* Linn, developed and registered by Natural Remedies Pvt. Ltd. (Bangalore, India). Following coarsely grinding, *C. longa* rhizomes were subjected to steamdistillation to remove volatile oil. The de-oiled plant material was subsequently extracted by aqueous reflux in a commercial manufacturing facility. To create a free-flowing powder, the liquid water extract was concentrated using vacuum distillation and then spray-dried. Turmeric oil and spray-dried water extract was combined in a 99:1 (w/w) ratio to create NR-INF-02, which was then sieved. The polysaccharide content of NR-INF-02 was determined to be 12.6% w/w by high- performance liquid chromatography. Curcuminoids content was negligible, as determined by a modified United States Pharmacopoeia (USP) method (19). Each batch produced was standardized to ensure consistent turmerosaccharide levels.

### 2.2 Animals

Twenty dogs were enrolled and randomly assigned to two treatment groups (*n* = 10 per group). Group I received the *C. longa* extract orally at a dose of 300 mg/20 kg body weight once daily for 42 days. Group II received a commercially available combination of glucosamine hydrochloride and chondroitin sulfate (Glu/CS) formulation orally for the same duration, administered according to the manufacturer’s recommendation. The study protocol was approved by the Institutional Animal Ethics Committee (IAEC) of Mumbai Veterinary College, Maharashtra Animal and Fishery Sciences University, India (Approval No. MVC/IAEC/06/2019) and conducted in accordance with the methodology approved by the PME Cell and IAEC-VCR.

### 2.3 Inclusion/exclusion criteria

Dogs were eligible for enrolment if they exhibited clinical signs of chronic lameness, stiffness, joint pain and had radiographic confirmation of OA. Animals receiving concurrent medications, pregnant bitches and dogs with lameness attributable to infectious causes or other diseases were excluded. Dogs with a history of prior pharmacological or nutraceutical treatment for arthritis were also excluded to avoid confounding effects (Table 12).

**Table 12:** Inclusion and exclusion criteria.

S. No.	Inclusion criteria	Exclusion criteria
1.	Dogs having clinical lameness (at least for 4 weeks) attributable to osteoarthritis due to previous trauma and aging, and surgically corrected joints.	Acute traumatic injuries
2.	Breeds preferred First preference: Labrador, Golden retrievers Second preference: Doberman, Bull mastiff, Great Dane	Exclusion breeds: Boxer and German Shepard
3.	Dogs older than 4 years with body condition score ≥2	Treatment with topical or systemic pharmaceuticals or biologics (other than routine anti-parasitic medication), corticosteroids, NSAIDs, or antimicrobials within 14 days before enrollment
4.	X-ray confirmation of osteoarthritis lesions (if considered necessary by the veterinarian)	Arthrocentesis/ intra-articular treatment with injectable depot corticosteroids; polysulfate glycosaminoglycan, glucosamine, or chondroitin sulfate nutritional supplements within 30 days before enrollment
5.	Absence of systemic illness	Animals which are pregnant; are receiving any medication; or have hepatic, renal or cardiovascular disease, gastrointestinal ulceration or a bleeding disorder will be excluded. Dogs with lameness due to infectious, immune-mediated, neurological or neoplastic disease and dogs which had received any previous drug or dietary supplement for the treatment for OA and dogs with overall score of clinical condition of very severely affected will also be excluded.

### 2.4 Health status examination

A full clinical examination was performed prior to the initiation of the experiment, including assessment of cardiovascular and systemic health. Radiographs of the affected joints were evaluated by an experienced veterinarian. The animals of same grade of severity of OA were selected for the study.

### 2.5 Assessment protocol

Clinical severity of each dog was recorded by the veterinarians at each visit and by the pet owners corresponding time points using ordinal and simple descriptive scoring system (Table 1, 2). Clinical scoring scale was developed based on subjective scoring systems as reported in earlier studies (26–28). Before initiation of the study, all owners were thoroughly briefed on the assessment protocol by one of the investigators to achieve the standardization of observation and analysis.

**Table 1:** Clinical scoring criteria used to score different outcome measures in dogs with osteoarthritis by veterinarians.

Criteria	Description	Score
Body condition score	Very thin	1
	Under weight	2
	Ideal	3
	Overweight	4
	Obese	5
Lameness	Walks normally (No Lameness)	1
	Slightly lame when walking	2
	Moderately lame when walking	3
	Severely lame when walking	4
	Reluctant to rise and will not walk more than five paces	5
Joint mobility impairment	Full range of motion	1
	Mild limitation (10–20%) in range of motion; no crepitus	2
	Mild limitation (10–20%) in range of motion; with crepitus	3
	Moderate limitation (20–50%) in range of motion; ±crepitus	4
	Severe limitation (>50%) in range of motion; ±crepitus	5
Pain on palpation	None (No Pain)	1
	Mild signs: dog turns head in recognition	2
	Moderate signs: dog pulls limb away	3
	Severe signs: dog vocalizes or becomes aggressive	4
	Dog will not allow palpation	5
Weight-bearing impairment	Equal on all limbs standing and walking	1
	Normal standing: favors affected limb when walking	2
	Partial weight-bearing standing and walking	3
	Partial weight-bearing standing; non-weight-bearing walking	4
	Non-weight-bearing standing and walking	5
Overall score of clinical condition (Average Score of B, C, D, and E)	Not affected	1
	Mildly affected	2
	Moderately affected	3
	Severely affected	4
	Very severely affected	5

**Table 2:** Clinical scoring criteria used to score different outcome measures in dogs with osteoarthritis by pet owners.

Criteria	Description	Score
Stiffness score		
How severe is your dog’s stiffness after first wakening in the morning?	None	1
	Mild	2
	Moderate	3
	Severe	4
	Extreme	5
Later in the day, how severe is your dog’s stiffness after lying down for at least 15 min?	None	1
	Mild	2
	Moderate	3
	Severe	4
	Extreme	5
How much of a problem does your dog have rising to standing after lying down for at least 15 min?	No problem	1
	Mild problem	2
	Moderate problem	3
	Severe problem	4
	Extreme problem	5
In general, over the past 7 days, how much difficulty has your dog had with his or her joints?	None	1
	Mild	2
	Moderate	3
	Severe	4
	Extreme	5
Function Score:		
Jumping up (as in getting into the car or onto the bed)?	No problem	1
	Mild problem	2
	Moderate problem	3
	Severe problem	4
	Extreme problem	5
Jumping down (as in getting out of the car or off of the bed)?	No problem	1
	Mild problem	2
	Moderate problem	3
	Severe problem	4
	Extreme problem	5
Climbing up (as in stairs, ramps or curbs)?	No problem	1
	Mild problem	2
	Moderate problem	3
	Severe problem	4
	Extreme problem	5
Climbing down (as in stairs, ramps, or curbs)?	No problem	1
	Mild problem	2
	Moderate problem	3
	Severe problem	4
	Extreme problem	5
General activity	No problem	1
	Mild problem	2
	Moderate problem	3
	Severe problem	4
	Extreme problem	5
Enjoyment of life	No problem	1
	Mild problem	2
	Moderate problem	3
	Severe problem	4
	Extreme problem	5
Gait score		
On average, how severe was your dog’s limp during mild activities (such as short walks)?	None	1
	Mild	2
	Moderate	3
	Severe	4
	Extreme	5
On average, how severe was your dog’s limp during moderate activities (such as long walks, playing or running)?	None	1
	Mild	2
	Moderate	3
	Severe	4
	Extreme	5
How often did your dog limp the day after moderate activities (such as long walks, playing or running)?	Never	1
	Rarely	2
	Occasionally	3
	Frequently	4
	Constantly	5
How often have you been aware of your dog’s joint problems?	Never	1
	Rarely	2
	Occasionally	3
	Frequently	4
	Constantly	5
Quality of life score		
In the past 7 days, what has been your level of concern that your dog is generally slowing down?	None	1
	Mild	2
	Moderate	3
	Severe	4
	Extreme	5
Overall, how would you rate your dog’s quality of life over the past 7 days?	Poor	1
	Fair	2
	Good	3
	Very Good	4
	Excellent	5

### 2.6 Treatment procedure

The selected dogs (*n* = 20) were randomized into two treatment groups (Group I and II). The dogs of Group I were assigned for the treatment with *C. longa* extract orally once a day at a dose of 300 mg/20 kg body weight for 42 days. The animals were observed post-treatment for 14 days. Thus, the total study period was 56 days. The dogs of Group II were treated with marketed drug for OA consisting of glucosamine and chondroitin sulfate as per recommended doses for 42 days and allowed for 14 days of post-treatment observation (Table 13).

**Table 13:** Details of the dogs included in the study.

Group	Case No.	Breed	Age (years)	Sex	Color	Weight (kgs)
I	1	Labrador	3.5	M	Black/Brown	38
	2	Lab x	11	F	Br/White	19
	3	Rottweiler	9	F	Bl/tan	45
	4*	Labrador	13	F	Golden	34
	5	Labrador	6	M	Fawn	32
	6	Lhasa	6	M	Tricolour	14
	7	GSD	5	M	Bl/Br	35
	8	Labrador	8	M	Fawn	43
	9	Labrador	7	M	Fawn	45
	10	Labrador	4	M	Black	35
Range		Lab/1 Lab x/1 Lhasa/1 GSD/1 Rot	3.5–13	(7 M/ 3 F)		14–45
II	1	GSD X	11	M	Bl/Br	27
	2	Labrador	12	M	Black	40
	3	ND	8	M	Black	22
	4	Pug	8	M	Light Br	9.7
	5	ND	7	M	Brown	20
	6	GSD X	12	M	Bl/Tan	28
	7	Rottweiler	4	F	Bl/Tan	29
	8	Labrador	10	M	Black	40
	9	GSD	10	M	B l/Tan	39
	10*	GSD	10	M	Bl/Tan	40
Range		GSD/2 GSD X/2	4–12	9M/1F		9.7–40

* The dogs were not included in final analysis due to owner non-compliance or comorbid conditions diagnosed during the study.

### 2.7 Assessment procedure

#### 2.7.1 Laboratory assessment

Blood samples were collected from the dogs in both treatment groups on day 0 and day 42 for hematological analysis. Serum was separated and stored at −20 °C for various biochemical parameters, including serum derivatives such as Prostaglandin E2 (PGE2) and cartilage oligomeric matrix protein (COMP). PGE2 concentrations were measured using a CUSABIO ELISA kit (Cat. No. CSB-E13493c), and COMP was quantified using a Cloud-Clone ELISA kit (Cat. No. SEB197Ca).

#### 2.7.2 Veterinary assessment

Veterinarians performed detailed examinations on days 0, 7, 14, 21, 28, 35, 42 and post-treatment days 49 and 56. Parameters evaluated included lameness, joint mobility, pain on palpation, weight bearing capacity, general condition and physiological variables (pulse rate, temperature, heart rate and mucous membrane color). Scoring was performed using a 5-point ordinal and descriptive scale (Table 1) (26–28).

#### 2.7.3 Owners’ assessment

The dog-owners were asked to complete a questionnaire (Table 2) about the changes in their pet’s well-being at the same time as the veterinary assessments. An ordinal and descriptive scale was used similar like veterinary assessments to rate physical condition, appetite, pain behavior and walking ability of the dogs, in addition to a Numerical Rating Scale to quantify the pain and overall satisfaction with the treatment regimen (26–28) (Table 2).

#### 2.7.4 Palatability assessment

Owners evaluated palatability and acceptability of the investigational products during the first 10 days of treatment using an established acceptability grading system (29–31) (Table 3).

**Table 3:** Acceptability grading system.

Criteria	Description	Score
A. Palatability	Excellent – Immediate Voluntary Reception	4
	Good – Hesitating Voluntary Reception	3
	Fair – Occasional Reluctant Reception	2
	Poor – Permanent Reluctant Reception (Forced to take)	1
B. Food Intake	Excellent – Takes full food	4
	Good – Takes 90% food	3
	Fair – Takes 75% food	2
	Poor – Takes 50% food	1

#### 2.7.5 X-ray

The radiographs of affected joints were obtained on days 0 and 42 and interpreted by radiologists at the Veterinary Hospital. Joint space narrowing, osteophyte formation and structural changes were assessed to evaluate progression of OA.

#### 2.7.6 Thermal imaging

Infrared thermal imaging of the affected joints was captured on day 0 and on day 42 using portable handheld infrared thermal camera (FLIR E6 IR camera Model no. 63902-0202). Images were interpreted by a veterinarian to assess changes in local temperature associated with inflammation.

### 2.8 Statistical analysis

Veterinary and owner assessment data are presented as Median (interquartile range, IQR) and analyzed using the Mann–Whitney test (SPPS version 21); results with *p* < 0.05 were considered statistically significant. Biochemical parameters are expressed as mean ± SEM and were analyzed using an unpaired *t*-test (GraphPad Prism 5); statistical significance was set at *p* < 0.05.

## 3 Results

In the present study, the efficacy of *C. longa* extract to treat OA in dogs of various breeds was evaluated and compared with a marketed product (Glu/CS). Initially 20 dogs (10/group) with similar grade of OA were selected, but in the final analysis a total number of 18 dogs (9/group) were included due to the comorbid conditions or non-compliance of the owner for the remaining 2 dogs.

### 3.1 Veterinarian assessment

Veterinarians evaluated the general body condition, lameness, joint mobility, pain on palpation, weight bearing and overall clinical condition of the animals at different time points in 7 days interval starting from day 0 (baseline) till day 42 (end of dosing) and 2 post-treatment time points, i.e., day 49 and 56. The results as mentioned in Table 4 showed significant improvement in lameness, weight bearing impairment and Overall scores of clinical conditions.

**Table 4:** Improvement of veterinarian clinical score at various time points

Clinical Parameters	Treatment groups	Day 0	Day 7	Day 14	Day 21	Day 28	Day 35	Day 42	Day 49	Day 56
Body condition score (A)	Group I (*Curcuma longa* extract)	3.0 (3.0–3.5)	3.0(3.0–3.5)	3.0(3.0–3.5)	3.0(3.0–4.0)	3.0(3.0–4.0)	3.0(3.0–3.0)	3.0(3.0–3.0)	3.0(3.0–3.0)	3.0(3.0–3.0)
	Group II (Glucosamine / Chondroitin sulfate)	3.0(2.5–3.5)	3.0(2.5–3.5)	3.0(2.5–3.5)	3.0(2.5–3.5)	3.0(2.5–3.0)	3.0(2.5–3.0)	3.0(2.5–3.0)	3.0(2.5–3.0)	3.0(2.5–3.0)
Lameness (B)	Group I (*Curcuma longa* extract)	3.0(3.0–3.5)	3.0(3.0–4.0)	3.0(3.0–3.5)	3.0(2.5–3.0)	3.0(2.0–3.0)	2.0(2.0–3.0)	2.0(2.0–2.0)***	2.0(1.5-2.5)***	2.0(1.5-2.5)***
	Group II (Glucosamine / Chondroitin sulfate)	3.0(3.0–3.5)	3.0(3.0–3.5)	3.0(3.0–3.5)	3.0(3.0–3.0)	3.0(2.0–3.0)	2.0(2.0–2.0)	2.0(2.0–2.0)^###^	2.0(2.0–2.0)^###^	2.0(2.0–2.0)^###^
Joint Mobility Impairment (C)	Group I (*Curcuma longa* extract)	3.0(2.0–3.5)	3.0(2.0–3.5)	3.0(2.0–3.5)	2.0(2.0–3.5)	2.0(2.0–3.5)	2.0(1.5–3.0)	2.0(1.0–2.5)***	2.0(1.0-2.5)	2.0(1.0–2.5)
	Group II (Glucosamine / Chondroitin sulfate)	3.0(2.0–4.0)	3.0(2.0–4.0)	3.0(2.0–4.0)	3.0(2.0–4.0)	2.0(2.0–3.5)	2.0(2.0–3.0)	2.0(2.0–3.0)	2.0(1.5–3.0)	2.0(1.0–3.0)
Pain on palpation (D)	Group I (*Curcuma longa* extract)	3.0(2.0–3.5)	3.0(3.0–3.5)	3.0(2.0–3.5)	3.0(2.0–3.0)	2.0(2.0–3.0)	2.0(2.0–3.0)	2.0(1.5–2.5)	2.0(1.5–3.0)	2.0(1.5–3.0)
	Group II (Glucosamine / Chondroitin sulfate)	2.0(1.5–4.0)	2.0(1.5–3.5)	2.0(1.5–3.5)	2.0(1.5–3.5)	2.0(1.5–3.5)	2.0(1.5–3.0)	2.0(1.5–2.5)	2.0(1.0–2.5)	2.0(1.0–3.0)
Weight Bearing impairment (E)	Group I (*Curcuma longa* extract)	4.0(2.5–4.0)	4.0(2.5–4.0)	4.0(2.5–4.0)	3.0(2.0–3.0)	3.0(2.0–3.0)	3.0(2.0–3.0)	2.0(1.0–3.0)***	2.0(1.0-3.0)***	2.0(1.0-3.0)***
	Group II (Glucosamine / Chondroitin sulfate)	3.0(2.5–3.5)	3.0(2.5–3.5)	3.0(2.5–3.5)	3.0(2.5–3.0)	3.0(2.0–3.0)	2.0(2.0–2.5)	2.0(2.0–2.0)^###^	2.0(1.5-2.0)^###^	2.0(1.5-2.0)^###^
Overall Score of clinical condition (Average of B, C, D and E)	Group I (*Curcuma longa* extract)	3.0(2.5–3.6)	3.0(2.5–3.7)	3.0(2.5–3.6)	2.5(2.2–3.2)	2.5(2.1–3.2)	2.2(1.7–2.8)	2.2(1.5–2.3)***	2.0(1.3-2.6)***	2.0(1.3-2.6)***
	Group II (Glucosamine / Chondroitin sulfate)	3.0(2.6–3.3)	3.0(2.5–3.3)	2.7(2.5–3.3)	2.7(2.3–3.2)	2.5(2.1–3.0)	2.0(2.0–2.5)	2.0(2.0–2.2)^###^	2.0(1.5-2.2)^###^	2.0(1.3-2.2)^###^

Values are expressed as Median (IQR); *n* = 9. Statistical analysis was carried out by Mann–Whitney Test using SPSS Version 21. ***p < 0.05 significantly different with the Day 0 of Group I. ^###^*p* < 0.05 significantly different with the Day 0 of Group II.

#### 3.1.1 Body condition score (BCS)

The median BCS in group I and II at the beginning of the study were 3.0(3.0–3.5) and 3.0(2.5–3.5) respectively. At the end of the treatment period (day 42), a non-significant decrease was noted in the BCS as 3.0(3.0–3.0) for group I and 3.0(2.5–3.0) for group II, indicating that the treatments did not affects general nutritional status.

#### 3.1.2 Lameness

The median lameness score in group I and II were 3.0(3.0–3.5) and 3.0(3.0–3.5) respectively on day 0 of assessment. A significant decrease in the lameness score was noted in group I from 28th day onwards up to day 42. However, a rebound increase in the lameness score was observed after treatment cessation (days 49 and 56), suggesting the improvement was treatment-associated. Owner reports corroborated the improvement in mobility.

#### 3.1.3 Joint mobility

The median joint mobility score at the beginning of the study in group I and II were 3.0(2.0–3.5) and 3.0(2.0–4.0) respectively. Improvement was noted in the joint mobility from 21st day onwards which continued up to day 42. However, an increase in the mobility score was noted on day 49 and 56 in both the groups.

#### 3.1.4 Pain on palpation

The median pain on palpation scores at the initial stage in group I and II were 3.0(2.0–3.5) and 2.0(1.5–4.0) respectively. Improvement was noted in the pain on palpation in group I from the 28th day onwards, which continued up to day 42. However, an increase in the score was noted on day 49 and 56 in both the groups after stopping the treatment with mild pain on palpation.

#### 3.1.5 Weight bearing impairment score

At the beginning, the median weight bearing impairment score in group I and II were 4.0(2.5–4.0) and 3.0(2.5–3.5) respectively. Group I exhibited significant improvement from day 21 to day 42, indicating better functional load bearing. Both groups showed increases in impairment scores after withdrawal.

#### 3.1.6 Overall clinical score

The median overall clinical score was calculated excluding the BCS in all the cases of the two groups. The mean overall score in group I and II were 3.0(2.5–3.6) and 3.0(2.6–3.3) respectively. A significant improvement was noted in the overall score in group I from the 28th day onwards, which continued up to day 42. However, an increase in the score was noted on day 49 and 56 in both the groups after stopping treatment.

### 3.2 Owners’ assessment

Owners were asked to complete a questionnaire about changes in their dog’s well-being at the same time points as the veterinary assessments and the physical condition, appetite, pain behaviors and walking ability of their pets were scored as mentioned in Tables 5–7.

**Table 5:** Mean score of owner’s description of stiffness at various time points.

Description of stiffness	Treatment groups	Day 0	Day 7	Day 14	Day 21	Day 28	Day 35	Day 42	Day 49	Day 56
Stiffness in the morning	Group I (*Curcuma longa* extract)	3.0(2.0–4.0)	3.0(2.0–4.0)	3.0(2.0–4.0)	3.0(1.5–4.0)	2.0(1.5–4.0)	2.0(2.0–4.0)	2.0(1.5–3.5)	2.0(1.5–3.0)	2.0(1.5–3.0)
	Group II (Glucosamine/Chondroitin sulfate)	2.0(2.0–3.5)	2.0(2.0–3.5)	2.0(2.0–3.5)	2.0(2.0–3.0)	2.0(2.0–3.0)	1.0(1.0–3.0)	1.0(1.0–2.5)	1.0(1.0–2.5)	1.0(1.0–2.5)
Stiffness after lying down	Group I (*Curcuma longa* extract)	3.0(2.0–4.0)	3.0(2.0–4.5)	3.0(2.0–4.5)	3.0(1.5–4.0)	2.0(1.5–4.0)	2.0(2.0–4.0)	2.0(1.5–2.5)	2.0(1.5–2.5)	2.0(1.5–2.5)
	Group II (Glucosamine/Chondroitin sulfate)	2.0(2.0–4.0)	2.0(2.0–4.0)	2.0(2.0–3.5)	2.0(2.0–3.0)	2.0(2.0–2.5)	1.0(1.0–3.0)	1.0(1.0–2.5)	1.0(1.0–2.5)	1.0(1.0–2.5)
Problem of standing after lying down	Group I (*Curcuma longa* extract)	3.0(2.0–4.0)	3.0(2.0–4.0)	3.0(2.0–4.0)	3.0(3.0–4.0)	3.0(2.0–4.0)	3.0(2.0–4.0)	2.0(1.5–3.0)	2.0(1.5–3.0)	2.0(1.5–3.0)
	Group II (Glucosamine/Chondroitin sulfate)	2.0(2.0–4.0)	2.0(2.0–4.0)	2.0(2.0–3.5)	2.0(2.0–3.0)	2.0(2.0–3.0)	1.0(1.0–3.0)	1.0(1.0–2.5)	1.0(1.0–2.5)	1.0(1.0–2.5)
Difficulty with the joints	Group I (*Curcuma longa* extract)	2.0(2.0–3.5)	2.0(2.0–3.5)	2.0(2.0–3.5)	2.0(2.0–3.0)	2.0(1.5–3.5)	2.0(1.5–3.5)	2.0(1.0–2.5)	2.0(1.0–2.5)	2.0(1.0–2.5)
	Group II (Glucosamine/Chondroitin sulfate)	3.0(3.0–4.0)	3.0(3.0–4.0)	3.0(3.0–3.5)	3.0(3.0–3.5)	3.0(2.0–4.0)	2.0(2.0–4.0)	2.0(1.0–3.0)^###^	2.0(1.0–3.0)^###^	2.0(1.0–3.0)^###^

Values are expressed as Median(IQR); *n* = 9. Statistical analysis was carried out by Mann–Whitney Test using SPSS Version 21. ***p < 0.05 significantly different with the Day 0 of Group I. ^###^*p* < 0.05 significantly different with the Day 0 of Group II.

**Table 6:** Mean score of owner’s description of function at various time points.

Description of function	Treatment groups	Day 0	Day 7	Day 14	Day 21	Day 28	Day 35	Day 42	Day 49	Day 56
Jumping up	Group I (*Curcuma longa* extract)	3.0(2.0–4.0)	3.0(2.0–4.0)	3.0(2.0–4.0)	3.0(2.0–4.0)	3.0(1.5–4.0)	3.0(1.5–3.5)	2.0(1.5–3.0)	2.0(1.0–2.5)	2.0(1.0–2.5)
	Group II (Glucosamine/Chondroitin sulfate)	2.0(2.0–4.5)	2.0(2.0–4.0)	2.0(2.0–4.0)	2.0(2.0–4.0)	2.0(2.0–4.0)	2.0(1.5–4.0)	1.0(1.0–3.5)	1.0(1.0–3.5)	1.0(1.0–3.5)
Jumping down	Group I (*Curcuma longa* extract)	3.0(2.0–4.0)	3.0(2.0–4.0)	3.0(2.0–4.0)	3.0(2.0–4.0)	3.0(1.5–4.0)	3.0(1.5–3.5)	2.0(2.0–3.0)	2.0(1.5–2.5)	2.0(1.5–2.5)
	Group II (Glucosamine/Chondroitin sulfate)	2.0(1.5–4.5)	2.0(1.5–4.5)	2.0(1.5–4.0)	2.0(1.5–4.0)	2.0(1.5–4.0)	2.0(1.5–4.0)	1.0(1.0–3.5)	1.0(1.0–3.5)	1.0(1.0–3.5)
Climbing up	Group I (*Curcuma longa* extract)	3.0(2.0–4.0)	3.0(2.0–4.0)	3.0(2.0–4.0)	3.0(2.0–4.0)	2.0(2.0–3.5)	2.0(2.0–3.5)	2.0(1.5–3.0)	2.0(1.0–3.0)	2.0(1.0–3.0)
	Group II (Glucosamine/Chondroitin sulfate)	2.0(1.5–4.0)	2.0(1.5–4.0)	2.0(1.5–4.0)	2.0(1.5–4.0)	2.0(1.5–4.0)	2.0(1.5–4.0)	1.0(1.0–3.5)	1.0(1.0–3.5)	1.0(1.0–3.5)
Climbing down	Group I (*Curcuma longa* extract)	3.0(2.0–4.0)	3.0(2.0–4.0)	3.0(2.0–4.0)	3.0(1.5–4.0)	3.0(1.5–4.0)	2.0(1.5–3.5)	2.0(2.0–3.0)	2.0(1.5–2.5)	2.0(1.5–2.5)
	Group II (Glucosamine/Chondroitin sulfate)	2.0(1.0–4.0)	2.0(1.0–4.0)	2.0(1.0–4.0)	2.0(1.0–4.0)	2.0(1.0–4.0)	2.0(1.0–3.5)	1.0(1.0–3.0)	1.0(1.0–3.0)	1.0(1.0–3.0)
General activity alteration	Group I (*Curcuma longa* extract)	3.0(2.0–4.0)	3.0(2.0–4.0)	4.0(2.0–4.0)	4.0(2.0–4.0)	3.0(2.0–4.0)	3.0(2.0–4.0)	3.0(2.0–3.0)	2.0(1.5–3.0)	2.0(1.5–3.0)
	Group II (Glucosamine/Chondroitin sulfate)	2.0(2.0–3.5)	2.0(2.0–3.5)	2.0(2.0–3.0)	2.0(2.0–3.0)	2.0(1.5–3.5)	2.0(1.5–3.5)	1.0(1.0–2.5)	1.0(1.0–2.5)	1.0(1.0–2.5)
Alteration in enjoyment of life	Group I (*Curcuma longa* extract)	2.0(1.0–3.0)	2.0(1.5–3.0)	2.0(1.5–3.0)	3.0(1.5–3.0)	2.0(1.5–3.0)	2.0(1.5–3.0)	2.0(1.5–2.5)	2.0(1.5–2.5)	2.0(1.5–2.5)
	Group II (Glucosamine/Chondroitin sulfate)	2.0(2.0–3.5)	2.0(2.0–3.5)	2.0(1.5–3.0)	2.0(1.5–3.0)	2.0(1.5–3.0)	2.0(1.5–2.5)	2.0(1.5–2.0)	2.0(1.5–2.0)	2.0(1.5–2.0)

Values are expressed as Median(IQR); *n* = 9. Statistical analysis was carried out by Mann–Whitney Test using SPSS Version 21. ***p < 0.05 Significantly different with the Day 0 of Group I. ^###^*p* < 0.05 Significantly different with the Day 0 of Group II.

**Table 7:** Mean score of owner’s description of gait and quality at various time points.

Description of gait and quality	Day	0	7	14	21	28	35	42	49	56
Limping during mild activity	Group I (*Curcuma longa* extract)	3.0(2.0–4.0)	3.0(2.0–4.0)	2.0(2.0–4.0)	2.0(2.0–4.0)	2.0(2.0–4.0)	2.0(2.0–3.5)	2.0(2.0–3.0)	2.0(1.5–3.0)	2.0(1.5–3.0)
	Group II (Glucosamine/ Chondroitin sulfate)	3.0(2.0–4.0)	3.0(2.0–3.5)	2.0(2.0–4.0)	2.0(2.0–4.0)	2.0(1.0–4.0)	2.0(1.0–3.5)	1.0(1.0–3-0)	1.0(1.0–3.0)	1.0(1.0–3.0)
Limping during moderate activity	Group I (*Curcuma longa* extract)	3.0(2.0–4.0)	3.0(2.0–4.0)	3.0(2.0–4.0)	3.0(2.0–4.0)	2.0(2.0–4.0)	2.0(2.0–3.5)	2.0(2.0–3.0)	2.0(1.5–2.5)	2.0(1.5–3.0)
	Group II (Glucosamine/ Chondroitin sulfate)	3.0(2.0–3.5)	3.0(2.0–3.5)	2.0(2.0–3.0)	2.0(2.0–3.0)	2.0(1.0–3.0)	2.0(1.0–3.0)	1.0(1.0–2.5)^###^	1.0(1.0–2.5)^###^	1.0(1.0–2.5)^###^
Limping day after moderate activity	Group I (*Curcuma longa* extract)	3.0(2.0–4.0)	4.0(2.0–4.0)	4.0(2.0–4.0)	3.0(2.0–4.0)	3.0(1.5–4.0)	3.0(1.5–3.5)	2.0(1.5–3.0)	2.0(1.5–3.5)	2.0(1.5–3.5)
	Group II (Glucosamine/ Chondroitin sulfate)	3.0(2.0–4.0)	3.0(2.0–3.5)	2.0(2.0–3.5)	2.0(2.0–3.5)	2.0(1.5–3.5)	2.0(1.5–3.5)	1.0(1.0–3.0)	1.0(1.0–3.0)	1.0(1.0–3.0)
Joint awareness	Group I (*Curcuma longa* extract)	3.0(2.0–4.0)	3.0(2.0–4.0)	3.0(2.0–4.0)	3.0(2.0–4.0)	3.0(2.0–4.0)	3.0(2.0–4.0)	3.0(2.0–3.5)	3.0(2.0–3.5)	3.0(2.0–3.5)
	Group II (Glucosamine/ Chondroitin sulfate)	2.0(2.0–4.5)	2.0(2.0–4.5)	2.0(2.0–4.5)	2.0(2.0–4.5)	2.0(2.0–4.5)	2.0(2.0–4.5)	2.0(2.0–4.0)	2.0(1.0–4.0)	2.0(1.0–4.0)
Level of prognosis	Group I (*Curcuma longa* extract)	4.0(3.0–4.0)	3.0(3.0–4.0)	3.0(3.0–4.0)	3.0(3.0–3.5)	3.0(3.0–3.5)	3.0(2.5–3.5)	3.0(2.0–3.0)***	3.0(1.5–3.0)***	3.0(1.5–3.0)***
	Group II (Glucosamine/ Chondroitin sulfate)	3.0(2.5–4.0)	3.0(2.5–3.5)	3.0(2.5–3.5)	2.0(2.0–3.5)	2.0(2.0–3.5)	2.0(2.0–3.5)	2.0(1.0–3.0)^###^	2.0(1.0–3.0)^###^	2.0(1.0–3.0)^###^
Quality of life	Group I (*Curcuma longa* extract)	3.0(2.0–3.0)	3.0(2.0–3.5)	3.0(2.0–3.5)	3.0(2.0–3.5)	2.0(2.0–3.5)	3.0(2.0–4.0)	3.0(2.0–4.0)	3.0(2.0–4.0)	3.0(2.0–4.0)
	Group II (Glucosamine/ Chondroitin sulfate)	3.0(2.0–3.0)	3.0(2.0–3.0)	3.0(2.5–3.5)	3.0(2.5–3.5)	3.0(2.5–3.5)	3.0(2.5–3.5)	3.0(2.0–3.5)	3.0(2.0–3.5)	3.0(2.0–3.5)

Values are expressed as Median (IQR); *n* = 9. Statistical analysis was carried out by Mann–Whitney Test with the aid of Graph Pad Prism Software version 5. ***p < 0.05 significantly different with the Day 0 of Group I. ^###^*p* < 0.05 significantly different with the Day 0 of Group II.

#### 3.2.1 Canine orthopaedic index

##### 3.2.1.1 Stiffness in the morning

The median stiffness in the morning score reported by owners at the beginning of the study in groups I and II was 3.0(2.0–4.0) and 2.0(2.0–3.5) respectively. A non-significant reduction in the score was noted in both the groups. However, though the decrease in stiffness was non-significant in group I, it was found to be better than the other group in terms of percentage of improvement.

##### 3.2.1.2 Stiffness after lying down for at least 15 min

The median score in the beginning in group I and II was 3.0(2.0–4.0) and 2.0(2.0–4.0) respectively. A significant decrease in the score was noted in group I while a non-significant decrease was noted in group II.

##### 3.2.1.3 Problem of standing after lying down

Initially, the median score in group I and II was noted as 3.0(2.0–4.0) and 2.0(2.0–4.0) respectively.

A significant decrease in the score was noticed in group I at various time intervals while a non- significant decrease was observed in group II. The decrease in the score in group I was to the extent of 11% on day 7, 18.67% on day 14, 22.33% on day 21, 26% on day 28 & 35, and 40.67% on day 42 which was better than the other group.

##### 3.2.1.4 Difficulty with the joints

At the initial stage, the median score in group I and II was observed as 2.0(2.0–3.5) and 3.0(3.0–4.0) respectively. A non-significant decrease in the score was noted in group I while a significant decrease was noted in group II at various time intervals. This non-significant decrease in the score in group I was to the extent of 7.33% on day 7, 11% on day 14, 18.67% on day 21, 22.33% on day 28, 26% on day 35 and 40.67% on day 42.

#### 3.2.2 Description of function

##### 3.2.2.1 Jumping up

Initially, the median score of jumping up activity recorded as 3.0(2.0–4.0) and 2.0(2.0–4.5) in group I and II, respectively. The decrease in the score was not insignificant in both the groups. The improvement was to the extent of 32.01% on day 21 & 28, 35.97% on day 35 and 39.93% on day 42 in group I, which was better when compared to the other group on day 21, 28, 35 and 42, respectively.

##### 3.2.2.2 Jumping down

The median score of jumping down in group I and II was 3.0(2.0–4.0) and 2.0(1.5–4.5) respectively at the initial stage of the study. The decrease in the score was not insignificant in both the groups. The improvement in group I was to the extent of 4.12, 12.73, 25.09, 29.21, 29.21, and 37.45% on day 7, 14, 21, 28, 35, and 42, respectively.

##### 3.2.2.3 Climbing up

The median preliminary score of climbing up in group I and II was 3.0(2.0–4.0) and 2.0(1.5–4.0) respectively. The decrease in the score was non-significant. The improvement was to the extent of 3.80% on day 7, 19.38% on day 14, 23.18% on day 21, 30.79% on day 28, 35 and 38.41% on day 42 in group I. A gradual improvement was observed in group I.

##### 3.2.2.4 Climbing down

Initially, the median score in group I and II was observed as 3.0(2.0–4.0) and 2.0(1.0–4.0) respectively. The decrease in the score was non-significant in both groups. The improvement to the extent of 4.12% on day 7, 16.86% on day 14, 25.09% on day 21, 29.21% on day 28, 35 and 33.33% on day 42 was noticed in group I. A gradual improvement was noted in group II on day 42 up to the extent of 35.97%.

##### 3.2.2.5 General activity alteration

The median score of general activity alteration at the beginning of the study in group I and II was 3.0 (2.0–4.0) and 2.0 (2.0–3.5) respectively. Decrease in the score was noted in group I at various time intervals indicating an improvement in the general activity of the animals. The improvement was to the extent of 10.61% on day 7, 21.54% on day 14, 28.62% on day 21, 32.15% on day 28, 35.69% on day 35 and 39.23% on day 42 in group I. A gradual improvement was noted in group I up to 49th day.

##### 3.2.2.6 Alteration in enjoyment of life

On day 0, the median score in group I and II was noted as 2.0(1.0–3.0) and 2.0(2.0–3.5) respectively. A non-significant decrease in the score was noted in both the groups at various time intervals indicating a general improvement in the enjoyment of life of the animals.

#### 3.2.3 Description of gait

##### 3.2.3.1 Limping during mild activity

Initially, the median score of limping in group I and II was noted as 3.0(2.0–4.0) and 3.0(2.0–4.0) respectively. Improvement in both the groups over the treatment duration. The percentage improvement in limping was to the extent of 3.53% on day 7, 10.61% on day 14, 17.68% on day 21, 21.54% on day 28, 28.62% on day 35 and 42.76% on day 42 in group I.

##### 3.2.3.2 Limping during moderate activity

At the beginning, the median score was found as 3.0(2.0–4.0) and 3.0(2.0–3.5) in group I and II, respectively. A non-significant decrease in the score was noted in group I while significant effect was observed in group II, indicating a general improvement in the limping during moderate activity.

##### 3.2.3.3 Limping day after moderate activity

Primarily, the median score in group I and II was 3.0(2.0–4.0) and 3.0(2.0–4.0) respectively. Non-significant decrease in the score was noted in group I and II. This indicated a general improvement in the limping after a day of moderate activity.

##### 3.2.3.4 Joint awareness

The median score in group I and II was 3.0(2.0–4.0) and 2.0(2.0–4.5) respectively at the beginning of the study. A significant decrease in the score was noted in group I at various time intervals while a non- significant decrease in the score was observed in group II. This revealed a general improvement in the joint awareness in group I.

##### 3.2.3.5 Level of prognosis

The median score of the level of prognosis at the initial stage in group I and II was 4.0(3.0–4.0) and 3.0(2.5–4.0) respectively. A significant decrease in the score was found in both the groups at various time points.

##### 3.2.3.6 Quality of life

The median score of quality of life in group I and II was 3.0(2.0–3.0) and 3.0(2.0–3.0) respectively.

A non-significant increase in the score was noted in groups I and II at various time intervals indicating a general improvement in the quality of life. The percentage improvement was better in group I on days 7, 14 and 21 as compared to the results found in group II.

#### 3.2.4 Palatability and food intake

The palatability and acceptability of the test products were found to be good to excellent and presented in Table 8. All the dogs willingly took the test products and the adverse effects like salivation, vomiting, diarrhoea and urticaria were not encountered during the test period.

**Table 8:** Palatability and food intake score.

Parameters	Group	Day 0	Day 5	Day 10
Palatability	Group I (*Curcuma longa* extract)	3.2 ± 0.21	3.3 ± 0.22	3.4 ± 0.16
	Group II (Glucosamine/Chondroitin sulfate)	3.4 ± 0.25	3.5 ± 0.25	3.5 ± 0.25
Food Intake	Group I (*Curcuma longa* extract)	3.6 ± 0.16	3.6 ± 0.16	3.6 ± 0.16
	Group II (Glucosamine/Chondroitin sulfate)	3.7 ± 0.25	3.7 ± 0.25	3.7 ± 0.25

The palatability and acceptability of both Group I and Group II were found to be good to excellent.

#### 3.2.5 Mean activity scores as per the fit bark collar

Three dogs in each group were provided with Fit bark collar for continuous monitoring of the pets with regards to their activity during and after the treatment period. A general improvement in the activity of the dogs was noted in all the dogs of both groups once the treatment was started, however, with stoppage of treatment a general decrease was again noted among all the animals.

### 3.3 Hemato-biochemical effects

The mean hematological values like Hb, RBC, PCV, WBC, neutrophils and platelet count of the animals of all groups were studied. No significant difference was noted in the parameters before and after the treatment (Table 9), which indicates the safeness of the product on the hematological parameters among the treated dogs.

**Table 9:** Hematocrit values before and after treatment.

Parameter	Normal reference ranges	Group I (*Curcuma longa* extract) (*n* = 6)		Group II (Glucosamine/Chondroitin sulfate) (*n* = 6)	
		Before	After	Before	After
Haemoglobin (gm %)	11.9–18.9	16.74 ± 0.93	16.13 ± 0.88 ^NS^	13.63 ± 1.09	14.41 ± 1.04 ^NS^
RBC (X 10^6^/cmm)	4.95–7.89	06.01 ± 0.41	06.42 ± 1.03 ^NS^	5.68 ± 0.13	5.55 ± 0.22 ^NS^
PCV (%)	35–57	41.3 ± 1.55	38.94 ± 2.06 ^NS^	37.61 ± 2.50	38.90 ± 1.56 ^NS^
WBC (X 10^3^/cmm)	5.0–14.1	11.71 ± 0.89	11.53 ± 0.91 ^NS^	13.37 ± 1.11	11.97 ± 0.94 ^NS^
Neutrophils (%)	58.0–85.0	78.6 ± 2.37	78.45 ± 2.13 ^NS^	77.01 ± 2.91	75.52 ± 3.07 ^NS^
Platelet (x10^5^/cmm)	2.11–6.21	2.43 ± 0.40	02.78 ± 0.29 ^NS^	2.44 ± 0.45	2.53 ± 0.37 ^NS^

Values are expressed as Mean ± SEM, *N* = 6, Statistical analysis was carried out by *t-*test with the aid of GraphPad Prism Software version 5. *p < 0.05 Significantly different with the group. NS, non-significant.

The means SGOT, SGPT, total protein, albumin, total bilirubin, direct bilirubin, alkaline phosphatase, serum creatinine and BUN were analyzed in the study (Table 10). A significant decrease was noted in the SGOT and serum creatinine levels, while a significant increase was marked in the albumin levels after treatment with the test drug in group I. No significant difference in the other parameters was noticed before and after treatment. In group II, there was a significant decrease noted in the alkaline phosphatase levels after treatment, however, no marked changes were noted in the mean SGOT, SGPT, albumin, total bilirubin, direct bilirubin, serum creatinine and BUN levels. The mean serum COMP and PGE2 levels were assessed among the studied animals of group I and II. There was no significant difference observed in COMP and PGE2 levels among the animals (Table 11).

**Table 10:** Biochemical values before and after treatment.

Parameter	Normal reference range	Group I (*Curcuma longa* extract) (*n* = 6)		Group II (Glucosamine/Chondroitin sulfate) (*n* = 6)	
		Before	After	Before	After
SGOT (IU/L)	18–56	68.43 ± 13.46	33.44 ± 7.89*	35.55 ± 15.82	36.25 ± 3.56^NS^
SGPT (IU/L)	17–95	50.46 ± 9.09	38.41 ± 5.53^NS^	43.50 ± 7.10	23.23 ± 3.99^NS^
TP (gm/dl)	5.4–7.5	7.42 ± 0.44	7.13 ± 038^NS^	6.89 ± 0.41	7.79 ± 0.62^NS^
AL (gm/dl)	2.3–3.1	2.68 ± 0.42	3.81 ± 0.26*	3.71 ± 0.44	4.17 ± 0.28^NS^
Total bilirubin (mg/dl)	0–0.3	1.24 ± 0.46	0.62 ± 0.16^NS^	0.54 ± 0.10	0.51 ± 0.07^NS^
Direct bilirubin (mg/dl)	0–0.3	1.07 ± 0.64	0.51 ± 0.10^NS^	0.40 ± 0.19	0.36 ± 0.06^NS^
Alkaline phosphatase (ALP) (IU/L)	7–115	130.47 ± 28.52	85.93 ± 17.7^NS^	91.94 ± 19.14	39.84 ± 8.98*
Serum creatinine (mg/dl)	0.5–1.7	1.00 ± 0.13	0.68 ± 0.10*	1.04 ± 0.17	1.01 ± 0.12^NS^
Blood urea nitrogen (BUN) (mg/dl)	8–28	33.69 ± 8.28	29.23 ± 2.66^NS^	25.34 ± 21.70	18.45 ± 10.38^NS^

Values are expressed as Mean ± SEM, *N* = 6, Statistical analysis was carried out by *t*-test with the aid of GraphPad Prism Software version 5. *p < 0.05 Significantly different with the group. NS = Non-Significant.

**Table 11:** COMP and PG-E_2_ values before and after treatment.

Parameter	Group I (*Curcuma longa* extract) (*n* = 6)		Group II (Glucosamine/Chondroitin sulfate) (*n* = 6)	
	Before	After	Before	After
COMP (ng/ml)	27.86 ± 5.60	39.01 ± 5.61 ^NS^	22.40 ± 3.71	20.12 ± 3.40 ^NS^
PG-E_2_ (pg/ml)	369.44 ± 191.71	536.47 ± 196.26 ^NS^	259.84 ± 105.03	293.77 ± 134.83 ^NS^

Values are expressed as Mean ± SEM, *N* = 6, Statistical analysis was carried out by *t-*test with the aid of GraphPad Prism Software version 5. *p < 0.05 significantly different with the group. NS, non-significant.

### 3.4 Evaluation with thermal camera

**Figure 1:**
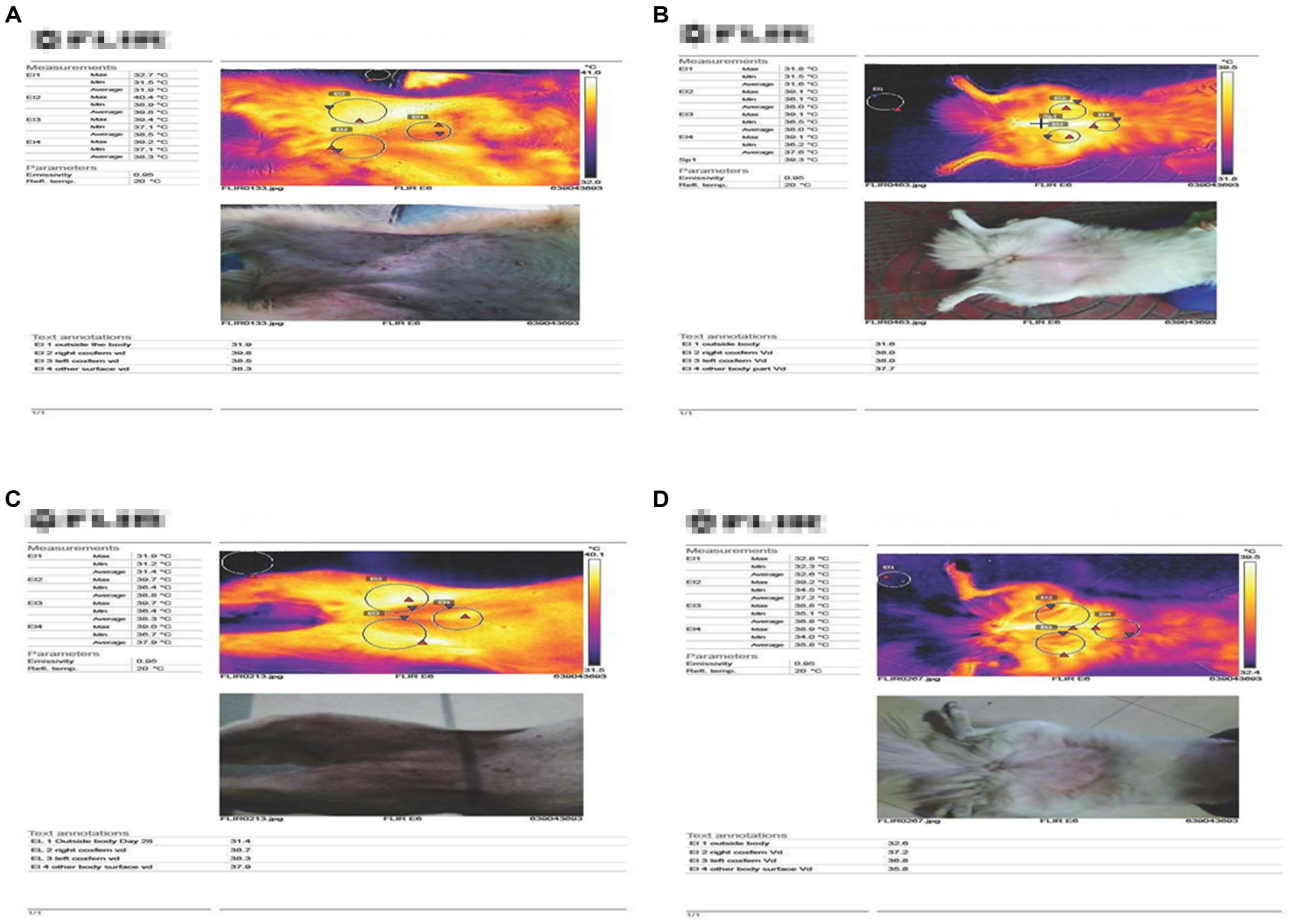
Evaluation with thermal camera. An increased temperature noted at the affected joint when compared with the other two surface temperatures **(A,B)**. However, with start of the treatment, in all the two groups the surface temperature at the affected joint was found to be decreased in **(C,D)**.

Whenever possible a few of the dogs were also assessed by thermal cameras in both the groups before and after treatment. The temperature using the thermal camera was taken outside the body surface, on the body surface and the affected joint. Thermal imaging demonstrated that affected joints exhibited higher surface temperatures than surrounding tissue at baseline. Both groups showed a decrease in joint surface temperature by day 42, consistent with reductions in inflammation. Temperatures rose again during the post-treatment period (Figure 1).

### 3.5 X-ray evaluation

**Figure 2:**
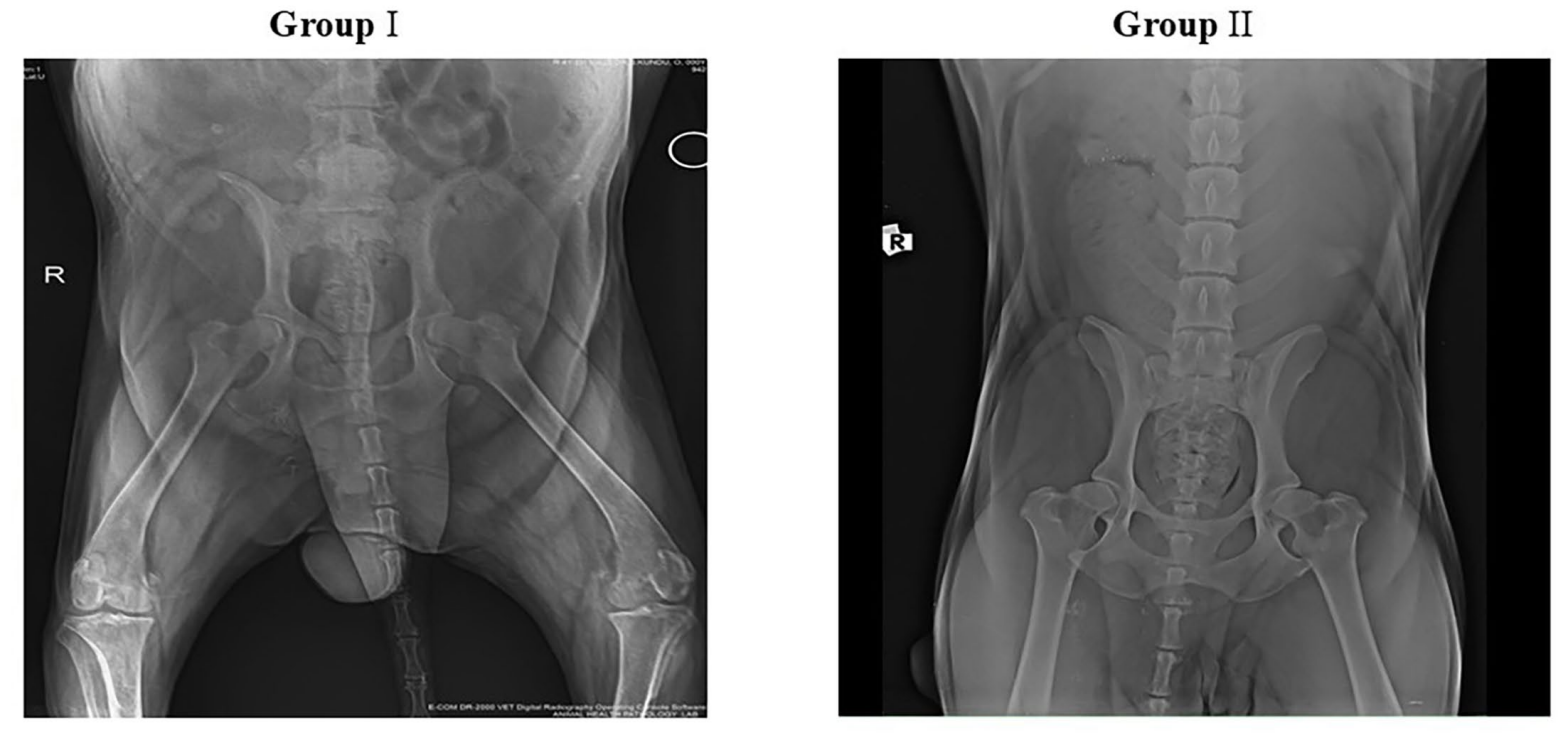
Radiography of osteoarthritis in dogs. Radiographs did not find any significant changes or worsening in the condition of the joints or the space between the bones within the joints in either of the two groups after 42 days of treatment.

Radiographs of the affected joints were taken just before treatment day 0 and the efficacy of treatment on the progression of OA lesions was evaluated on day 42. Radiographs obtained on days 0 and 42 revealed no major alterations in joint structure or joint-space width in either group. These findings suggest that neither treatment contributed to measurable radiographic progression of OA over the study period (Figure 2).

## 4 Discussion

Osteoarthritis is a progressive musculoskeletal disorder characterized by degenerative changes of the articular cartilage ultimately leads to subchondral bone abrasion, synovitis, joint stiffness and pain. As OA is irreversible, identifying an effective therapy to alleviate clinical symptoms is essential (32). In the present trial, we conducted a comparative evaluation of the therapeutic efficacy of *C. longa* extract with the marketed formulation for the treatment of OA in dogs. Animals were treated for 42 days and monitored for an additional 14-day post-treatment period. OA upsets the normal well-being of dogs by introducing pain and discomfort among the animals. Considering ethical responsibility, a placebo control group was not included. The experiment result showed a significant improvement in the overall score of clinical condition of the dogs in both groups I (*C. longa* extract) and II (marketed drug) for OA. Although, the percentage improvement was noticed better in group I than in group II as assessed by veterinarians. Group I demonstrated a significant decrease in scores like joint mobility impairment, pain on palpation and weight bearing impairment. However, scores in both groups increased following treatment cessation.

The subjective assessment through questionnaires for dog owners further supported the clinical findings, indicating improvement in stiffness and general activity in both group I and group II. However, the percentage recovery was better in group I as compared to group II. Significant improvement in the limping during mild activity, joint awareness, level of prognosis and quality of life was noted at various time intervals. The percentage of improvement in case of joint awareness and level of prognosis was higher in group I. Thermal imaging revealed reduced surface temperature in affected joints in both groups, indicating decreased local inflammation. Radiographic assessments suggested a slowing of joint deterioration in response to both interventions.

The findings of this study are aligned with previous reports demonstrating the beneficial effects of curcuminoid-containing diets on OA-associated pain in dogs. In a randomized, double-blind, prospective, placebo-controlled trials the diet containing curcuminoids extract benefited from arthritic pain in OA animals (33). In another double- blinded, randomized, crossover, placebo- controlled trial for 10 weeks, the client-owned dogs with mild to moderate OA were treated with the dietary mixture containing curcumin (bioactive molecule derived from *C. longa*). The study showed positive results in reducing the joint pain and lameness among the animals according to the owners’ observations (2, 32). According to the investigations based on the experiences of veterinary practitioners, glucosamine and chondroitin sulfate exhibit chondroprotective effect. Pharmacodynamics, pharmacokinetic, safety and efficacy study data further support their beneficial effect in managing canine OA. However, further research is required to determine the most effective dosage of these compounds (34). In a double blind, randomized, positive controlled, multicenter clinical trial lasting 70 days on dogs, treatment with chondroitin sulfate/glucosamine hydrochloride resulted in improved overall clinical score as assessed by veterinarian (1).

Both the test and reference products were well tolerated, with satisfactory palatability. The data obtained from hemato-biochemical analysis in group I showed no major deviation from normal values. Although serum PGE2 and COMP levels in group I and II did not differ significantly between groups. In the current trial the increased levels of PGE2 and COMP in Group I suggest persistent inflammation and continuous cartilage turnover, while the decline in these markers in Group II suggests better regulation of these pathways following the intervention. The non-significant results for the objective biomarkers may have occurred due to higher inter-individual variability or insensitivity to subtle treatment effects, whereas the significant improvements in subjective scores represent real symptomatic relief, mediated by central or psychosomatic effects of the therapy. Such discrepancies are common in chronic conditions, where the improvement perceived by the patient is often reported much earlier before changes in objective molecular markers are detected. Previously, Fujiki et al. reported that Polysulfated glycosaminoglycan (PSGAG) treatment can alter the COMP levels and improve the lameness score in OA affected dogs (35). They demonstrated that intramuscularly dosing of PSGAG prevented COMP degradation in OA affected dogs. Jerosch et al. summarized the chondroprotective effect of nutraceutical, especially antioxidants along with other nutrients like glucosamine, chondroitin sulfate, hyaluronic acid, collagen hydrolysate can modulate OA with an excellent safety profile (36).

The extract of *C. longa* containing bioactive compounds like turmerosaccharides is well studied for its anti-inflammatory activity. It acts through various pathways that impedes the OA pathogenesis. Chandrasekaran et al. demonstrated immune-stimulatory and anti-inflammatory activities of turmerosaccharides through *in vitro* studies on mouse splenocytes and mouse macrophages (19). They demonstrated a significant increase in Nitric oxide (NO), Interleukin (IL) − 2, IL-6, IL-10, IL- 12, interferon gamma (IFN *γ*), tumor necrosis factor alpha (TNF*α*) and Monocyte chemoattractant protein-1 (MCP-1) production in the unstimulated mouse splenocytes and macrophages resulting in, immune-stimulatory activity. Mechanistic studies in human chondrocytes further indicate protection against the release of Prostaglandin E2 (PGE2) and IL-12 levels in lipopolysaccharide (LPS) stimulated mouse splenocytes thereby exhibiting the anti-inflammatory activity (19). In a chondroprotective mechanistic study, turmerosaccharides significantly inhibited the IL-1β-induced chondrocyte death and apoptosis and release of chondrocyte degradation markers such as IL-6, IL- 8, Cyclooxygenase (COX) 2, PGE2, TNF-α, Intercellular Adhesion Molecule 1 (ICAM-1) in human chondrocyte (NHAC-kn cells) and attenuated LPS-induced NF-κB expression in RAW264.7 cells. Additionally, turmerosaccharides also protect against IL-1β-induced damage to glycosaminoglycans, type II collagen and further inhibited H2O2-induced chondrocyte senescence thereby protected cartilage homeostasis in chondrocytes by maintaining the equilibrium between anabolic and catabolic factors of cartilage matrix (37).

Human clinical studies corroborate these findings, demonstrating significant improvements in pain and joint function following administration of *C. longa* extracts, including formulations standardized to turmerosaccharides. Madhu et al. reported a significant improvement in the Visual Analogue Scale (VAS), Western Ontario and McMaster Universities Osteoarthritis Index (WOMAC) scale and Clinician Global Impression Change (CGIC) scale post treatment with *C. longa* extract in OA patients (22). Raj et al. reported that *C. longa* extract was effective when compared to placebo in increasing the pain threshold and knee range of movement (ROM) in healthy participants (38, 39). Wang et al. reported that aqueous *C. longa* extract standardized to turmerosaccharides in combination with curcuminoids significantly reduces the knee pain and improves joint function among people with inflammatory knee osteoarthritis (40). In another study, water- soluble Turmeric extracts and insoluble curcuminoids formulation reduced self- reported, mild/moderate joint pain after 3 days and 1 week of treatment. The analgesic effect of turmerosaccharides was progressive followed by a decrease in inflammatory status which suggests that it could be safely used as an emergency painkiller for human knee joint pain (41). Selvi et al. reported that extract of *C. longa* is safe and tolerable at till 2 g administered in healthy adult volunteers (42).

In our present trial, no adverse events were observed in dogs treated with *C. longa* extract. In agreement with previous studies, we did not find any major changes in hemato-biochemical parameters during the study (43–45). Although the reference product was also effective, glucosamine and chondroitin have been associated with gastrointestinal disturbances and minor hematological changes in previous studies, warranting further investigation into their long-term safety (1). Further studies are needed to determine to evaluate their toxicity after long term use of glucosamine and chondroitin sulfate. Moreover, our study data exhibited that the overall improvement of the OA condition in dogs of group I treated with *C. longa* extract was better than the dogs of group II administered with reference product. A limitation of the current study is the relatively small sample size, which may restrict generalizability and reduce the statistical power to detect between-group differences. Larger, controlled studies are required to confirm these findings and further delineate mechanisms of action.

## 5 Conclusion

This clinical trial exhibited that *C. longa* extract is at least as effective as a marketed glucosamine– chondroitin formulation in improving clinical signs of OA in dogs, with superior outcomes in pain on palpation, weight-bearing capacity, and functional activity. Further significant improvements across multiple subjective and objective parameters, together with a favorable safety profile, support the therapeutic utility of *C. longa* extract at a dosage of 300 mg/20 kg once daily in canine OA. Thermal imaging also corroborated reductions in joint inflammation during treatment. These findings highlight *C. longa* extract as a promising, well-tolerated intervention for managing osteoarthritis in dogs.

## Data Availability

The original contributions presented in the study are included in the article/supplementary material, further inquiries can be directed to the corresponding author/s.
